# MicroRNA-like RNAs from the same miRNA precursors play a role in cassava chilling responses

**DOI:** 10.1038/s41598-017-16861-w

**Published:** 2017-12-07

**Authors:** Changying Zeng, Jing Xia, Xin Chen, Yufei Zhou, Ming Peng, Weixiong Zhang

**Affiliations:** 1 The Institute of Tropical Bioscience and Biotechnology, Chinese Academy of Tropical Agricultural Sciences, Haikou, 571101 China; 2 Institute for Systems Biology, Jianghan University, Wuhan, Hubei 430056 China; 3 Department of Computer Science and Engineering, Washington University in St. Louis, St. Louis, MO 63130 USA; 4 Department of Genetics, Washington University School of Medicine, St. Louis, MO 63130 USA

## Abstract

MicroRNAs (miRNAs) are known to play important roles in various cellular processes and stress responses. MiRNAs can be identified by analyzing reads from high-throughput deep sequencing. The reads realigned to miRNA precursors besides canonical miRNAs were initially considered as sequencing noise and ignored from further analysis. Here we reported a small-RNA species of phased and half-phased miRNA-like RNAs different from canonical miRNAs from cassava miRNA precursors detected under four distinct chilling conditions. They can form abundant multiple small RNAs arranged along precursors in a tandem and phased or half-phased fashion. Some of these miRNA-like RNAs were experimentally confirmed by re-amplification and re-sequencing, and have a similar qRT-PCR detection ratio as their cognate canonical miRNAs. The target genes of those phased and half-phased miRNA-like RNAs function in process of cell growth metabolism and play roles in protein kinase. Half-phased miR171d.3 was confirmed to have cleavage activities on its target gene P-glycoprotein 11, a broad substrate efflux pump across cellular membranes, which is thought to provide protection for tropical cassava during sharp temperature decease. Our results showed that the RNAs from miRNA precursors are miRNA-like small RNAs that are viable negative gene regulators and may have potential functions in cassava chilling responses.

## Introduction

MicroRNAs (miRNAs) form a class of endogenous, 20–22nt long regulatory RNA molecules. They exert their function of post-transcriptional gene regulation through mRNA cleavage, RNA degradation, and translation inhibition^1,2^. Most canonical miRNAs are transcribed by RNA polymerase II (Pol II) to produce pri-miRNA transcripts, which are then cleaved by RNase III-type enzymes called Dicer-like proteins into stem-loop structured precursors in the nucleus^3^. Stem-loop pre-miRNAs are subsequently cleaved into miRNA/miRNA* duplexes by Dicer or Dicer-like enzymes in the cytoplasm^4,5^. The mature miRNAs are then incorporated into ARGONAUTE (AGO)-containing RNA-induced silencing complexes (RISC) in the cytoplasm to exert their regulatory effects by guiding the RISC to target transcripts through perfect or partially complementary base pairing^6^.

Non-canonical microRNAs have been discovered in various organisms^7–13^. Non-canonical miRNAs have structural and function similar with canonical miRNAs, but they can skip one or more steps of classic miRNA biogenesis pathway^7^. Mirtrons were firstly discovered Drosha/Dgcr8 independent and intron splicing can generate pre-miRNA-sized hairpin substrates for the following Dicer cleavage^8^. Small nucleolar RNA-derived miRNAs^9^, endogenous short hairpin RNAs derived miRNAs^10^ and tRNA-derived miRNA^11^ are three Dicer-dependent, Dgcr8-Independent miRNAs. Surprisingly, Dicer-independent pathways have also been identified. For example, simtron^12^ and miR451^13^ can interact with Ago and are found to be Drosha-dependent and Dicer-independent functional miRNAs. The diverse origins of non-canonical miRNAs help amend and broaden our understanding of miRNA biogenesis.

Typically, one miRNA/miRNA*duplex is expected to arise from a miRNA precursor. With the growing popularity of Next Generation sequencing of small RNAs, mapping of sequencing reads to genome sequences has often resulted in many non-negligible small RNA reads beyond miRNAs and miRNA*s, which can align perfectly to the “stem” regions of pre-miRNA precursors. Some of these small RNA species even present in high abundance. Multiple distinct miRNA-like RNAs can arise from a single miRNA precursor and they have been reported in plant species and mammals^14–17^. Some of these miRNA-like RNAs have been shown to be authentic and functional miRNAs: e.g. de-suppression of the targets was observed in *dcl1–9* mutants which produced no miRNA-like RNAs, and negative correlation between the expression of miR477a.3 and their targets (At1g54710 and At1g06770) was confirmed under the challenge of *Pst* (avrRpt2) infection where miR477a.3 was highly induced^15^. In addition, miR319b.2 were non-canonical miRNAs generated from *Arabidopsis* pri-miR319, and it was proven to be a functional miRNA targeting RAP2.12 mRNA during inflorescences development^18,19^.

Plant miRNA precursors often comprise several alternative fold-back structures with highly variable sizes and shapes, range from 50- to 900-nt^9,20^. Bologna *et al*.^21^ and Schapire *et al*.^22^ systemically analyze miRNA processing intermediates during the biogenesis of small RNAs in *Arabidopsis*, by using an approach called Specific Parallel Amplification of RNA Ends (SPARE). They found that at least 4 different processing pathways are involved in the generation of most miRNAs in *Arabidopsis*. Diverse long-stem RNA structures of pre-miRNA and different competing RNA domains might generate two different small RNAs by recognizing alternative miRNA determinants. The above method has revealed an unexpected complexity of the miRNA processing pathways acting upon both ancient and evolutionarily young sequences, and shown that members of the same miRNA family can be produced in different ways.

Cassava is a tropical staple crop that provides an essential source of diet for over a half billion of people in developing countries. Cassava is prone to cold injuries during spring planting and autumn harvest. Most previous studies of cassava miRNAs focus mainly on miRNA identification by computational analysis using cassava EST and GSS database^23,24^ and by next-generation sequencing of small RNAs from specific tissues^25,26^. The functions of miRNA-like RNAs in response to cold stress remain under explored in plants, especially in cassava. Our previous studies profiled and analyzed miRNAs and endo-siRNAs response to four different chilling treatments^27,28^. Remapping of the small-RNA sequencing reads revealed phased and half-phased miRNA-like RNAs originating from many miRNA precursors. Here we report our findings of the newly discovered phased and half-phased miRNA-like RNAs and the results of experimental validation of these miRNA-like RNAs, and their possible functions in cassava cold responses.

## Results

In the current study, we fully utilized the small-RNA deep sequencing profiling data that we collected from cassava that were subjected to four cold treatments^27^ (see Methods). Two cassava cultivars, SC124 and C4, were used in this study, where SC124 is chilling sensitive and subject to small RNA sequencing, and C4 is more chilling tolerance and used in experimental validation.

### Phased miRNA-like RNAs

**Figure 1:**
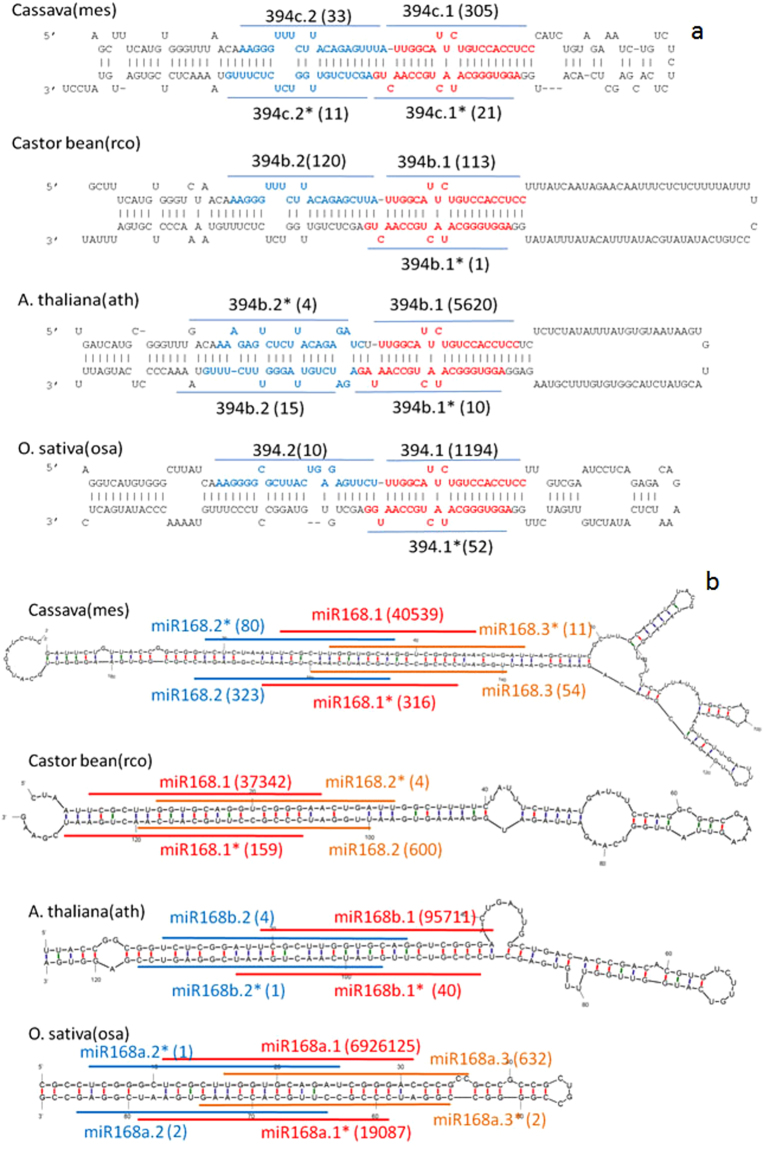
Examples of phased miRNA-like RNAs in cassava and castor bean and their conservation in Arabidopsis and rice^15^. (**a**) From miRNA394c precursor. (**b**) From miRNA168 precursor. A number in parenthesis next to a miRNA name is the number of reads of the miRNA or miRNA-like RNA.

It has been shown that many *Arabidopsis* miRNA precursors (pre-miRNAs) can yield multiple distinct ~21-nt miRNAs in phasing along the same precursors^15^, many of which are conserved in other plants, including rice, Poplus, Medicago and Moss^15^. Six and four pre-miRNAs in cassava and castor bean, respectively, have this property (Table S1A, Fig. 1a and Figure S1). Take miRNA394, a conserved plant miRNA, as an example. Besides the major miR394, a second miRNA-like RNA adjacent to the major miRNA was arranged in phase on pre-miR394 in cassava and castor bean (Fig. 1a). A similar phasing pattern was observed on pre-miRNA394 in *Arabidopsis* and rice (Fig. 1a). Following the naming convention^11^, we called the canonical mature miRNA miR394c.1 and the additional miRNA-like RNA miR394c.2. Another example is pre-miR169 which is conserved in these four species (Figure S1A). The analysis of the sequencing data also identified 4 and 1 novel miRNA-like RNAs in cassava and castor bean, respectively (Figure S1A). The expressions of six phased miRNA-like RNAs were experimentally validated by qRT-PCR. Particularly, miR394c.2, #28.2 and #40.2 were detected along with their major miRNAs (Table 1 and Figure S5). Note that the qRT-PCR detection rate of phased miRNA-like RNAs is similar to that of the corresponding canonical miRNAs, both of which are around 50%. These results indicated that there exist Euphorbiaceous miRNA precursors with certain features recognized by Dicer, and the mechanism for generating multiple miRNAs from one pre-miRNA is conserved in Euphorbia.

**Table 1:** Summary of qRT-PCR confirmation in cassava.

Assay	Selected miRNA number in validation experiment (24)	
Type	confirmed	not confirmed
Canonical miRNA	5/10 (50%)*	5/10 (50%)
	#40, #54, miR394abc, miR168.1, miR390b.1	#28, #44ab, #60.1, miR169b.1, miR171hi
Phase miRNA	3/6 (50%)	3/6 (50%)
	miR394c.2, #28.2, #40.2	miR169b.2, miR394b.2, #60.2
	7/8 (83.3%)	1/8 (16.7%)
Half-phase miRNA	miR166b.2, miR168.2, miR168.3, miR171d.2, miR171d.3, miR390b.2, #54.2	#44a.2
Total	15/24 (62.5%)	9/24 (37.5%)

*The numerator and denominator represent the detected miRNA number and total assayed miRNA number. The percentage of detection ratio by qRT-PCR shown in bracket.

### Half-phased miRNA-like RNAs

While there exist miRNA isoforms that shift by 1- to 3-nt from the 5′ ends of major miRNAs^29^, cassava and castor bean may also have miRNA-like RNAs that deviate by 4- to 11-nt from the 5′ ends of major miRNAs, which are unlikely miRNA isoforms. We named these new miRNA-like RNAs as half-phased miRNA-like RNAs^30,31^. Reflected by the sequencing data, 9 and 3 pre-miRNAs in cassava and castor bean, respectively, could produce half-phased miRNA-like RNAs (Figure S1B). Examples of half-phased miRNA-like RNAs include cassava pre-miR168 and pre-miR390b are shown in Fig. 1b and Figure S1B, respectively. Furthermore, half-phased miRNA-like RNAs miR166b.2, miR168.2, miR168.3, miR171d.2, miR171d.3, miR390b.2 and #54.2 were experimentally validated (Table 1 and Table S5). It is important to note that the detection rate of half-phased miRNA-like RNAs is significantly higher than that of canonical miRNAs (83% vs. 50%).

Most of these half-phased miRNA-like RNAs had their miRNA*-like sequences with ~2-nt 3′-overhangs (Fig. 1b and Figure S1B), showing perfect miRNA/miRNA* duplexes produced by endonuclease Dicer processing. This property of half-phased miRNA-like RNAs is conserved beyond Euphorbia, as shown by the examples of half-phased miRNA-like RNAs derived from miR168′s precursor in *Arabidopsis* and rice (Fig. 1b).

### Phased and half-phased miRNA-like RNAs are differentially expressed

**Figure 2:**
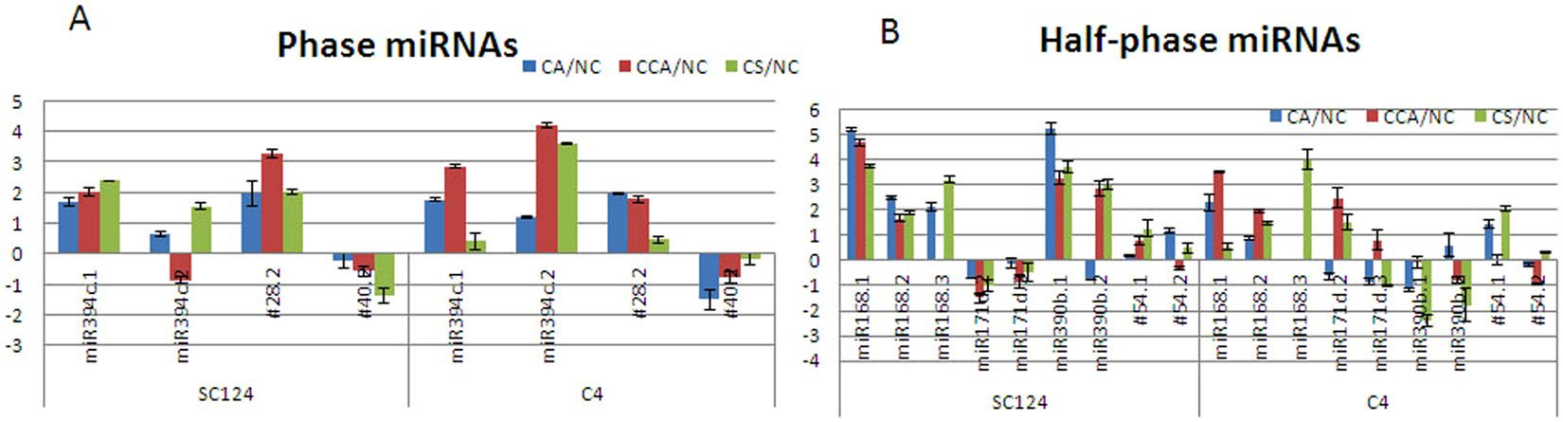
Experimental validation of differential expression of (**A**) phased miRNAs-like RNAs and (**B**) half-phased miRNAs-like RNAs under three cold stress treatments, CA, CCA and CS, comparing to the normal condition in cassava cultivars SC124 and C4.

We identified six highly abundant phased miRNA-like RNAs in cassava in total (Table 1A), three of which (miR394c.2, #28.2 and #40.2) were validated to be cold responsive (Fig. 2a, Table S5). Remarkably, some miRNAs and their phased miRNA-like RNAs had different expression patterns in the two cassava cultivars. For example, the expression patterns of miR394c.1 and miR394c.2 differed between SC124 and C4 in response to cold stress (Fig. 2a). MiR394c.1 was highly up-regulated under mild cold acclimation (CA: 14 °C) and harsh cold treatment after cold acclimation (CCA: 4 °C after 5 days treatment of 14 °C) in both cultivars, whereas miR394c.2 exhibited no response to mild cold treatments in SC124. A more remarkable difference was observed between #28.1 and the phased #28.2. Specifically, #28.1 was not detected while #28.2 was significantly up-regulated under all three cold conditions in the two cassava cultivars.

Nine half-phased miRNA-like RNAs were also differentially expressed as detected in sequencing data, six of which were subject to further qRT-PCR analysis (Fig. 2b and Tables S5). Similarly, different expression patterns between canonical miRNAs and their half-phased miRNA-like RNAs were also detected between the two cassava cultivars. For instance, the degrees of up-regulation of miRNA-168 (i.e., miRNA-168.1) and the two half-phased miRNA-like RNAs from pre-miR168 (i.e., miR-168.2 and miR-168.3) were all greater in SC124 than in C4 under the three cold treatments. However, miR390b.2 was highly up-regulated in SC124 but sharply down-regulated in C4 under the cold shock treatment (CS: sharply drop from 24 °C to 4 °C) in comparison to the normal condition (Fig. 2b).

Furthermore, 13 PCR products from some known miRNAs, novel miRNAs, phased miRNA-like RNAs and half-phased miRNA-like RNAs were retrieved, cloned and sequenced to examine their specificity and to ensure the authenticity of qRT-PCR experiment. Ten sequences out of the total 13 tested PCR amplicons were the same as the expected sequences (Table 2), indicating a reasonably high specificity of the qRT-PCR experiment.

**Table 2:** Sequence validation of miRNA, phased and half-phased miRNA-like RNAs by PCR amplicon sequencing.

Type	miRNA	Sequence of RNA-seq	Sequence by PCR cloning	validation
Novel	ARF1	TTCTTGACCTTGTAAGACCTT	TTCTTGACCTTGTAAGA	∽
Novel	ARF2	TTCTTGACCTTGTAAGACCCC	TTCTTGACCTTGTAAGACCCC	√
Novel	D8+	CCATACTTTGAATTGTGGAAG	CCATACTTTGAATTGTGGAAG	√
Known	miR168.1	TCGCTTGGTGCAGGTCGGGAA		×
Half-phase	miR168.2	TGCATCAACTGAATCGGAGAC	TGCATCAACTGAATCGGAGAC	√
Half-phase	miR168.3	TGGATCCCGCCTTGCATCAAC	TGGATCCCGCCTTGCATCAAC	√
Half-phase	miR171d.2	AGATATTGGTGCGGTTCAATC		×
Half-phase	miR171d.3	TTGTTGTTTGATTGAGCCGTG	CACGGCTCAATCAAACAACAA	√
known	miR390b.1	AAGCTCAGGAGGGATAGCGCC	GGCGCTATCCCTCCTGAGCTT	√
Half-phase	miR390b.2	CAGGAGGGATAGCGCCATGGA	TCCAGTCGCTATCCCTCCTG	∽
Known	miR394c.1	TTGGCATTCTGTCCACCTCC		×
Phase	miR394c.2	AAGGGTTTCTTACAGAGTTTA	AAGGGTTTCTTACAGAGTTTA	√
Known	miR398	TGTGTTCTCAGGTCGCCCCTG	CAGGGGCGACCTGAGAACACA	√

“√”, “∽” and “×” means that the sequence of PCR products is as same as, nearly as same and not as same as the sequence determined by RNA-seq, respectively.

In summary, the existence of phased and half-phased miRNA-like RNAs indicated that the miRNA biogenesis and gene regulation are more diverse and broader than previously understood.

### Differentially expressed miRNAs regulating their target mRNAs under cold stress

**Figure 3:**
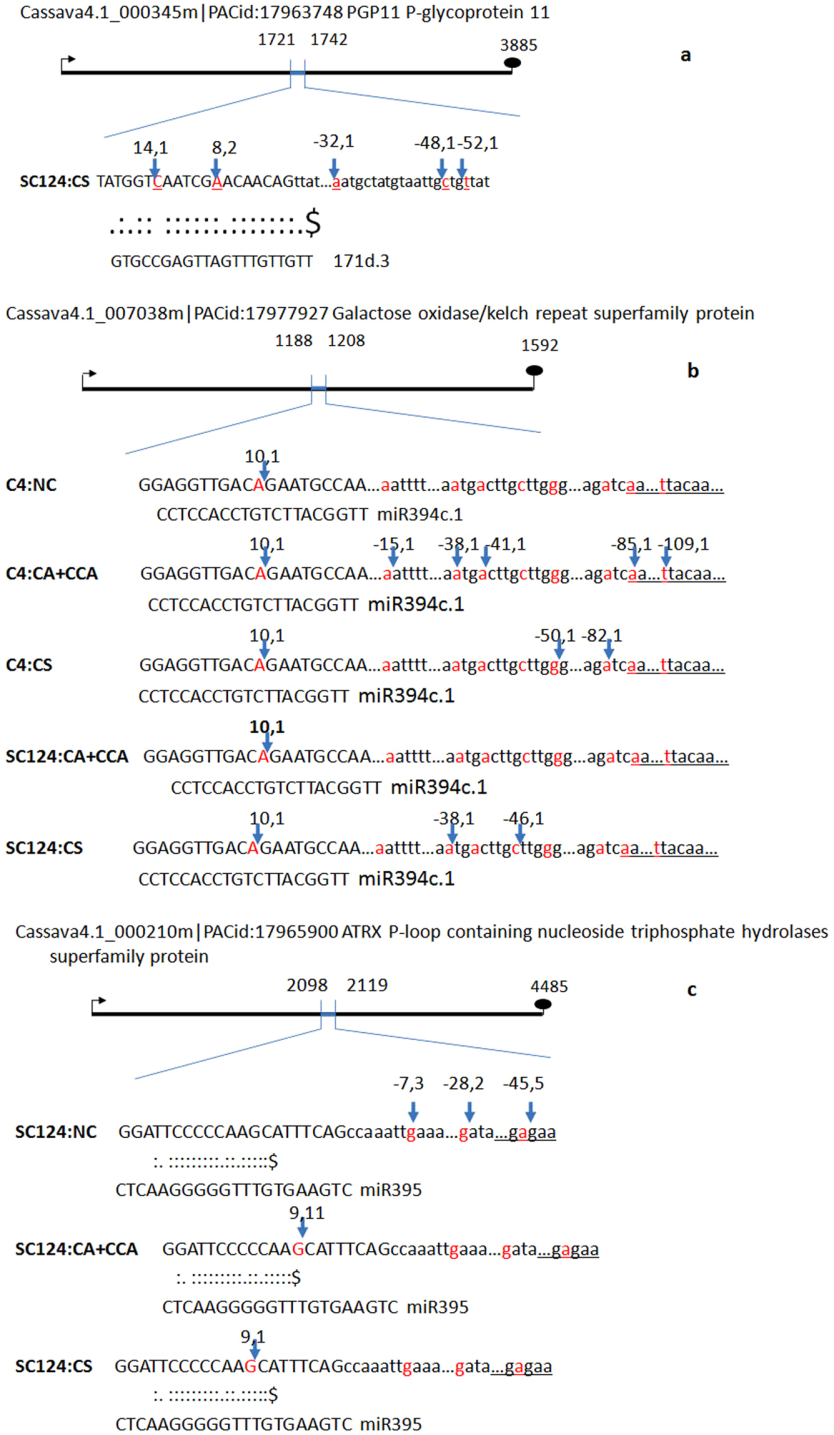
MiRNA cleavage sites on target genes of miR395, miR394 and half-phased miR171d.3 identified by 5′-RACE analysis. For each miRNA, the target sequence is shown on the top and the miRNA sequence on the bottom. The numbers above the arrow indicate the position and events of cloned PCR products. (**a**) The cleavage site of cassava4.1_000345m by miR1171d.3. (**b**) The cleavage site of cassava4.1_007038m by miR394c.1. (**c**) The cleavage site of cassava4.1_000210m by miR395. SC124 and C4 are two cassava cultivars used in this study, and NC, CA, CCA and CS are the four cold treatments (see methods).

Besides the existence of qRT-PCR and target prediction of these miRNA-like RNAs, more important is their potential functions as post-transcriptional gene regulators. Indeed, some of the phased and half-phased miRNA-like RNAs detected here had their own mRNA targets that were different from the targets of major miRNAs (Table S3). Analysis of miRNA target cleavage activities by 5′RACE is widely adopted to provide direct experimental evidence of regulatory function of miRNAs. We selected 8 miRNA:target pairs for further experimentally analysis and validation of cleavage of target mRNA transcripts. Six target mRNA transcripts showed evidences of direct cleavage: three of them were cleaved within the base-pairing region, 1 of them was cleaved at the upstream of the base-pairing region and 5 of them were cleaved at the downstream of miRNA complementary regions (Fig. 3 and Table S6). In addition to validating the cleavage of transcripts by canonical miRNAs (such as miR395, miR394c.1 and miR390b.1), half-phase miRNAs (miR171d.3 and miR390b.2) were also shown to trigger cleavage of their own targets (Fig. 3 and Table S6). This indicated that the newly discovered half-phased miRNA-like RNAs could regulate the expression of their own target genes.

The targets of those phased and half-phased miRNA-like RNAs were predicted (Table S3). These target genes function mainly in metabolism processes of cell growth, protein phosphorylation, dephosphorylation, and transmembrane transportation.

## Discussion

With improved sequencing capabilities and analysis, new types of small noncoding RNAs are continuously identified and experimentally validated. In addition to canonical miRNA, a number of non-canonical pathways for miRNA biogenesis have also been described^32–34^. A common feature of these non-canonical pathways is the cleavage of intermediate RNA precursors by Dicer-like or AGO2 or Drosha proteins^31,35,36^. In contrast to animal miRNAs, small RNA machineries and alternative miRNA pathways for miRNA processing in plants require further investigation. Thanks to the prevalence of next generation deep-sequencing technologies, comprehensive quantification of the small RNA transcriptome becomes a routine procedure, which makes it feasible to systematically profile and study distinct phased small-RNAs originating from miRNA precursors beyond the canonical mature miRNA or miRNA* duplex.

Biogenesis pathways for non-canonical miRNA production have been reported^7–13^. Nevertheless, most of them were discovered using mutants of some key components of canonical miRNA biogenesis, such as *Dicer*, *Drosha*, *dgcr8*, *and Ago*. As for tropical cassava, this approach, unfortunately, is currently not effective since such mutants are not available in Euphorbia. The phased and half-phased miRNA-like RNAs reported here are specific and limited to classic stem structures of pre-miRNAs, which differ from isomiRs (at the same miRNA positions) and moRNAs (flanking sequence beyond pre-miRNAs) in pig subcutaneous fat miRNome^37,38^, and varied from isomiRNAs and phased siRNAs (not miRNAs) in cotton small RNA^39^. The number of reads mapped to phased and half-phased miRNAs is comparable to or even greater than the number of reads mapped to mature miRNA or miRNA* sequences. It remained to be studied regarding how these additional small RNAs are generated and what functions they serve *in planta*. It has been suggested that large terminal loops in pri-miRNAs readily trigger non-canonical launching of a microprocessor, resulting in alternative processing^31,40,41^.

Bologna *et al*.^20^ found that miR319 precursors have large fold-back sequences and undergo a non-canonical processing pathway. Briefly, the process of the precursors begins with the first cleavage below the terminal loops, and continues with three more dicing events until the mature miRNAs are released. The first two cleavage activities potentially generate small RNAs, and miR319a.2/b.2/c.2 are subsequently generated from the upper part of the same hairpin structures. Sobkowiak *et al*.^19^ further provided evidence that the first cleavage produces a non-canonical functional miRNA (also called miR319b.2) which targets RAP2.12 intron-retained isoform during plant senescence and plant response to osmotic stress.

Besides triggered by native miRNAs, miRNA-like RNAs can be produced in artificial miRNA (amiRNA) transgenic plants. Guo *et al*.^42^ and Carbonell *et al*.^43^ demonstrated that many miRNA-like RNAs could be accumulated in the artificial miR319a-precursor transgenic Petunia and OsMIR390-transgenic rice. The production of non-canonical miRNAs should be taken into account when investigating the accuracy, specificity and efficiency of amiRNA-induced gene silencing technology used in genetic improvement.

Little was known about miRNA-like RNAs from a single miRNA precursor in cassava, and differed from the previous computational analysis. The current study relied heavily on experimental assay. We analyzed small RNAs detected by high-throughput small RNA sequencing in four chilling treated cassava. Seven miRNA-like RNAs from MIR genes (miR168, miR171, miR390, miR394, #28, #40, and #54) were qRT-PCR assayed. Their amplification products were then cloned and sequenced to validate the results of small-RNA deep sequencing. These experiments confirmed the identities and abundances of phased and half-phased miRNA-like RNAs detected through the profiling of cassava small-RNA transcriptome. These phased and half-phased miRNA-like RNAs have a comparable detection rate as canonical miRNAs and exhibit expression variations in response to different chilling treatments. The target genes of those miRNA-like RNAs mainly function in metabolism processes, including protein kinase activity, protein dephosphorylation, transmembrane movement of substances, proteolysis and methyltransferase activity, which help cassava use physiological adjustment of growth and development to adapt to cold stress. Furthermore, 5′ RACE results confirmed that half-phased miRNA-like RNA miR171d.3 could act upon PGP11, a target gene different from the targets of canonical miR171d. Gene PGP11 belongs to the superfamily of ATP-binding cassette (ABC) transporters, which pumps many foreign substances out of cells across extra- and intra-cellular membranes, and evolve as a defense mechanism against harmful substances in animals, fungi and bacteria^44^. MiR171d.3 is down-regulated in both cultivars under cold shock treatment, which exerting a negative impact on tropical cassava when temperature sharply declined from 24 °C to 4 °C. In this case, target cleavage of miR171d.3 specifically occurred in SC124 but not in cold-tolerant C4; that PGP11 was not cleaved by miR171d.3 in C4 might help protect C4 from harmful damage during cold exposure. Take together, the non-canonical miRNA-like RNAs will enhance the canonical miRNA-mediated gene regulation and add a new dimension to our understanding of complex gene regulatory networks.

## Methods

### Small RNA profiling of cassava chilling stress acclimation by NextGen Sequencing

Four distinct chilling stress treatments of cassava cultivar SC124 were administrated, and small RNAs from the plants were sequenced as described in former transcriptome paper^27^. Briefly, the four cold treatments include 1) gradual chilling acclimation (CA) where plants grown in the normal condition of 24 °C were stressed to 14 °C for 5 days; 2) chilling stress after chilling acclimation (CCA) where plants after 5 days of the CA treatment were transferred further from 14 °C to 4 °C for another 5 days; and 3) chilling shock (CS) where subjected normal plants to a dramatic temperature drop from 24 °C to 4 °C to reached 4 °C at the same time as the CCA treatment. For comparison, plants grown continuously under 24 °C were used as the normal control (NC).

Three tissues – folded leaf, fully expanded leaf and roots of cassava cultivar SC124 –were harvested at 6 h, 24 h and 5d for the three chilling treatments of CA, CCA and CS as well as the normal condition as described above. Tissue’s total RNAs were isolated separately, and equal amount of total RNAs from 3 time points were then pooled together for expression profiling in given tissue. Similarly, three tissues samples pooled equally. As a result, four small-RNA libraries of SC124, corresponding to the conditions of CA, CCA, CS and NC, were constructed.

In parallel, two chilling treatments upon castor bean (NC and CCA) were carried at the same time. In total, six small-RNA libraries (four for cassava and two for castor bean) were subjected to small-RNA deep sequencing by Illumina Genome Analyzer (GAIIx).

### Identifying miRNA-like RNAs and determining their phasing patterns

We first mapped sequence reads, allowing no mismatches, to miRNA precursors. We retained those precursors that have reads arranged in block patterns for further analysis. To this end, we applied the Blockbluster software^45,46^ to first identify blocks of sequencing reads on the annotated miRNA precursors. The most abundant sequence read within each detected block was taken as the representative sequence for the block. The total number of sequence reads for the block is the sum of the copy number of the representative read, those of other sequence reads that fall within the representative sequence and that overlap with the representative read with no more than three nucleotides beyond the representative sequence on either end. We allowed such overhangs to tolerate imprecise dicing activities of RNase III enzymes and account for possible miRNA isoforms^29^. The remaining blocks were further inspected; those spanning across two neighboring blocks were ignored. Those miRNA precursors that have blocks of reads arranged in phase were kept for further analysis. When reporting the results using our cassava deep sequencing data, we ignored such blocks that have less than five total sequencing reads to reduce potential false positive miRNA-like RNAs due to sequencing error.

### Expressional and sequencing validation of phase and half-phase miRNAs-like RNA

To further validate and characterize miRNA-like RNAs, a cold-tolerance cultivar, C4, was used in subsequent experimental assays. To profile expression patterns, a multiplexed RT method was applied to assess the expression of selected differential expressed miRNAs. Total RNA was first-strand cDNA synthesized with pool of miRNA-specific RT primers. These RT primers contain unique tag sequences at the 5′-ends and 7- to 10-nt complementary nucleotides with 3′ ends of specific miRNAs. Real-time PCR was then performed with the cDNA templates generated from the multiplexed RT reaction. The PCR reverse primer specifically anneals to the 5′-end of the cDNA templates, and the PCR forward primer specifically anneals to the unique tag sequence used in the RT primer. The forward and reverse primers were designed following the strategy by Wang^47^, which was developed to amplify mature miRNAs. The amplicons included 21- to 25-nt miRNA specific primers and 30-nt adaptors designed for the common reverse primer template, resulting in ~55-nt length target. The sequences of PCR primers are listed in Table S2.

Real-time PCR was performed following the standard SYBR Premix Ex TaqTM kit (TaKaRa) protocol. The reactions were incubated in 0.1 ml tubes of Rotor-gene 6000 machine, and the melt-curve was used to test the specificity of the tested PCR products. A negative control (no template) was included for each primer combination.

Gene U6 served as a reference for miRNA in each sample, and genes were amplified in parallel for 3 replicates. The relative concentration was calculated as 2 powered − ΔΔCT, where ΔΔCT = (ΔCT sample − ΔCT control), ΔCT = CT (target) − CT (reference) in each sample. If the CT value was greater than that of no template control (NTC), the miRNA was considered not expressed.

The ~55-nt amplified PCR products were then gel purified, cloned and sequenced (Sangon, China).

### The cleavage sites detected by 5′ RACE

RNA ligase-mediated rapid amplification of 5′cDNA ends (RLM-RACE) GeneRacer Kit (Invitrogen, USA) was used to validate miRNA-guided mRNA cleavage, which differed with traditional 5′RACE of full-length cDNA by omitting the 5′ phosphates of truncated mRNA removal and the 5′ cap structure of full-length mRNA removal treatments. Briefly, total RNA was extracted with RNAplant regent (TIANGEN, DP407-01), and Poly(A) RNA was isolated using polyAtract mRNA isolation system III (Promega, USA) to eliminate contaminated non-mRNA. Ligation with a 5′ RNA adapter and a reverse transcription were performed. The resulting cDNA was used as a template for PCR amplification. Two ~100 bp spaced gene specific reverse primers (GSP1 and GSP2) for each target, designed based on the downstream sequence of the miRNA:target binding site at the target gene sequence. Combining with two GeneRacer 5′ forward primers (included in GeneRacer kit) to specifically nest amplify the 3′ cleavage product of the target mRNA. The amplified PCR products were gel purified, cloned, sequenced (Sangon, China), and the cleavage site determined by align sequenced data to target gene. The target prediction of phased and half-phased miRNA-like RNAs is listed in Table S3, and gene specific primers that we used are provided in Table S4.

## Electronic supplementary material

Supplemental Figure S1

Supplemental Tables
