# Supplementary material for: MicroRNA-like RNAs from the same miRNA precursors play a role in cassava chilling responses
Source: Sci Rep. 2017 Dec 7;7:17135. doi: 10.1038/s41598-017-16861-w (PMC5719433; doi:10.1038/s41598-017-16861-w)

MicroRNA-like RNAs from the same miRNA precursors play a role in Cassava chilling responses

Changying Zeng[1,#], Jing Xia[2#], Xin Chen[1], Yufei Zhou[1], Ming Peng[1,†], Weixiong Zhang[3,†]

Supplemental Figure S1. Examples of phased miRNA-like RNAs from (A) miRNA169 and (B) half-phased miRNA-like RNAs from miR390b hairpins miRNA169bhairpins in Cassava and Castor bean as well as their conservation in rice and Arabidopsis]. A number in parenthesis next to a miRNA name is the number of reads of the miRNA.

(A)

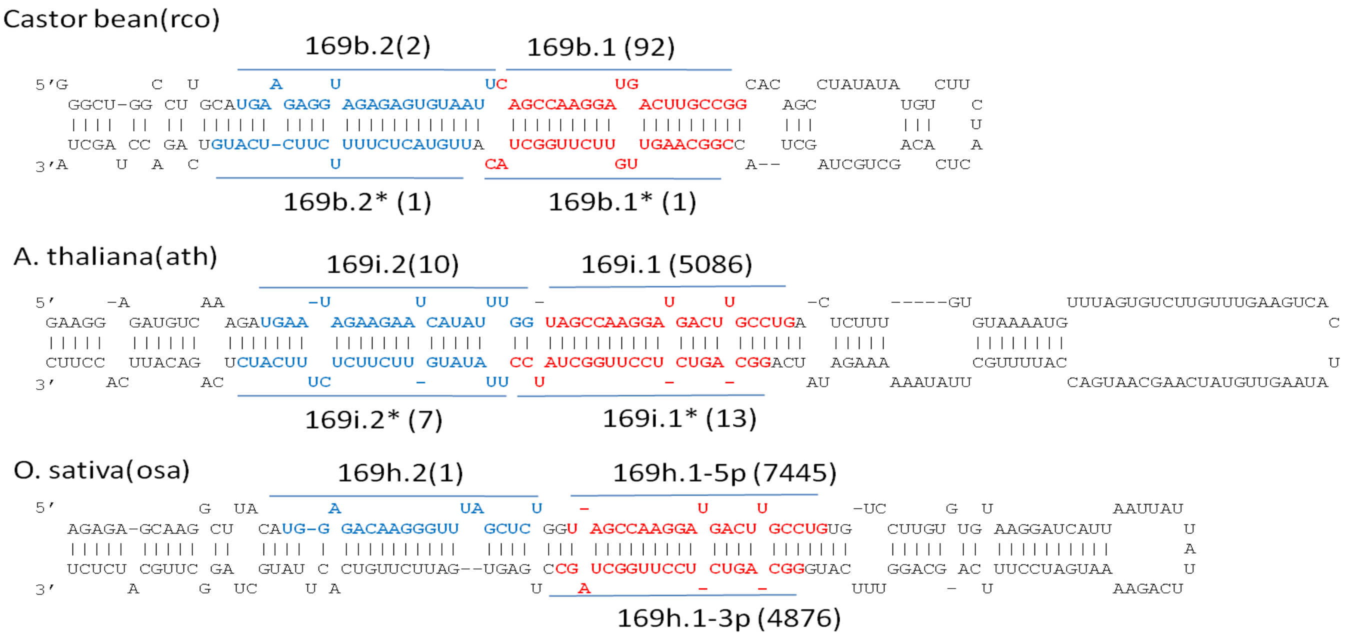

(B)

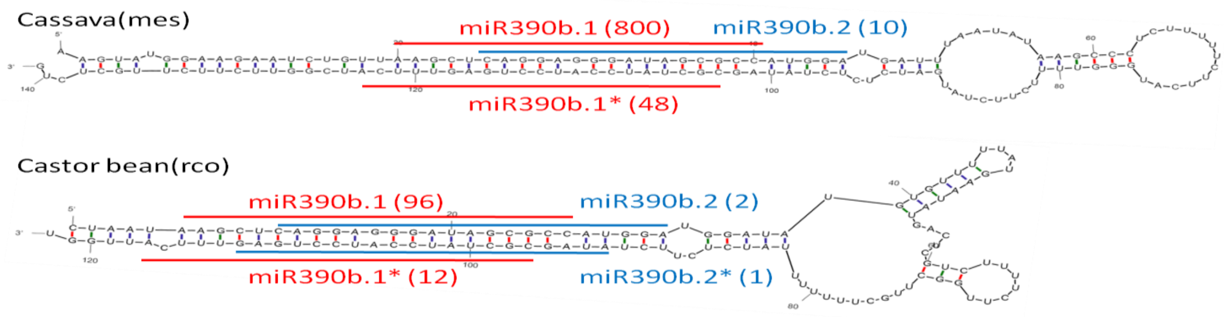

Supplement: Supplementary file 1 — Supplemental Figure S1 [file 41598_2017_16861_MOESM1_ESM.pdf]
